# Treatment of tongue cavernous haemangioma with direct puncture and sclerotization with ethanol

**DOI:** 10.2478/raon-2014-0006

**Published:** 2015-03-03

**Authors:** Tomaz Seruga, Jernej Lucev, Marko Jevsek

**Affiliations:** Radiology Department, University Medical Centre Maribor, Slovenia

**Keywords:** vascular abnormalities, cavernous haemangioma, direct puncture, ethanol, oil contrast media

## Abstract

**Background:**

Haemangiomas of tongue are rare type of malformations. They can be treated mostly conservatively but in some cases they need more aggressive treatment with preoperative intra arterial embolization and surgical resection. Lesions of tongue that are localized superficially can also be treated with direct puncture and injection of sclerosing agent (absolute ethanol).

**Case report:**

We present a case of a 48 years old female patient, where we performed embolization of cavernous haemangioma with mixture of absolute ethanol and oil contrast. After the procedure the patient received analgetics and antioedematous therapy. After the sclerotization the planed surgery was abandoned. Control MRI examinations 6 and 12 months after the procedure showed only a small remnant of haemangioma and no signs of a larger relapse.

**Conclusions:**

In our case the direct puncture of haemangioma and sclerotherapy with ethanol proved to be a safe and effective method to achieve preoperative devascularization of the lesion. Direct puncture of the lesion is not limited by the anatomy of the vessels or vasospasm, which can occur during the intra-arterial approach.

## Introduction

Vascular malformations of the tongue are relatively rare disorders. According to Mulliken and Glowacki they are divided into haemangiomas and vascular malformations (Table 1).^1^

The nomenclature in this field is very problematic, particularly in assessing benign vascular anomalies. Currently a variety of systems adapted for and by distinct clinical subgroups, like pathologists, clinicians, radiologist, are in use. In 1982, Mulliken and Glowacki proposed a binary classification system (Table 1) for vascular anomalies based on pathologic features.^1^ These system was adopted by the International Society for the Study of Vascular Anomalies (ISSVA) and has since been expanded and is now widely accepted. The importance of the ISSVA system is that it allows a systematic approach to vascular lesions that correlates predictably with clinical history, disease course, and treatment options, making it clinically useful.^2^

The ISSVA classification system divides vascular anomalies into 2 primary biological categories (Table 2). The major distinction between the 2 categories is whether there is increased endothelial cell turnover, which is ultimately determined by the identification of mitoses seen on histopathology. Vasoproliferative neoplasms have increased endothelial cell turnover. Vascular malformations do not have increased endothelial cell turnover. Instead, vascular malformations are structural abnormalities of the capillary, venous, lymphatic, and arterial system that grow in proportion to the child.^2^ Although ISSVA classification system is widely accepted, other nomenclatures are in use, but none of the current classification schemes are universally accepted. That continues to cause confusion, misunderstood diagnoses, and potential mismanagement. In order to avoid excessive entanglement, we decided to use Mulliken and Glowacki classification in our article, because detailed classification for the understanding of the article is not necessary.

Haemangiomas are the most common tumour of infancy and typically appear as a small reddish macule. About 80% occur within the first month of life. The macule quickly grows and becomes raised and lobulated. Majority of haemangiomas involve the skin, they can occur subcutaneously, appearing as a bluish patch under the skin. This can cause a rapidly developing swelling which then involutes as in the cutaneous lesion.

The term vascular malformations (VM) refer to lesions where the anatomy and morphology of the vessels are abnormal although the vascular endothelium is normal. These lesions can either be high, low or mixed flow lesions.

Classification that considers also clinical symptoms is named after Schobinger and has four stages, where stage 1 presents as a blue-skin blush, stage 2 as a mass associated with a bruit and a thrill, stage 3 as a mass associated with ulceration, bleeding and pain and stage 4 as a stage 3 lesions producing heart failure.^1^

Each vascular abnormality requires a serious diagnostic approach. Based on the findings the most appropriate method of treatment is selected.^3^ Location of the lesion, its topography, the size and geometry can be best shown by the contrast enhanced magnetic resonance imaging (MRI).^4^ Next diagnostic method is a digital subtractional angiography (DSA), which can show us the angioarchitecture of the malformations and flow dynamic within the lesion.

The invasive treatment of the tongue vascular abnormalities is usually the preoperative endovascular embolization followed by the surgical resection of the lesion or partial resection of the tongue, depending on the size of the lesion.^5^,^6^ Preoperative embolization is preformed because intraoperative bleeding can significantly influence the outcome of the surgical treatment. Intra-arterial catheter embolization can be technically difficult because of the complex vascular anatomy and the vasospasm.^5^ Problems connected with the intra-arterial catheter embolization can be solved by direct puncture and injection of alcohol into the lesion. In doing so, care must be taken that the injection of the embolization material is limited to the lesion and that there is no recourse to the venous side of the malformation.^7^ The success of the therapy is evaluated clinically, later follow up is provided by MR examinations.^1^

## Case report

A 48 years old female patient was admitted to our institution because of the difficulties in swallowing food and the swelling of the tongue. The otorhinolaryngology exam showed bluish, localized, approximately 35 × 25 mm big swelling on the upper side of tongue, with vessels shining through the glossal mucosa. The lesion was localised on the right side of the tongue in the middle part. The radix of the tongue was free. After the biopsy the histological examination of the tissue was performed and cavernous haemangioma was diagnosed.

**FIGURE 1.**
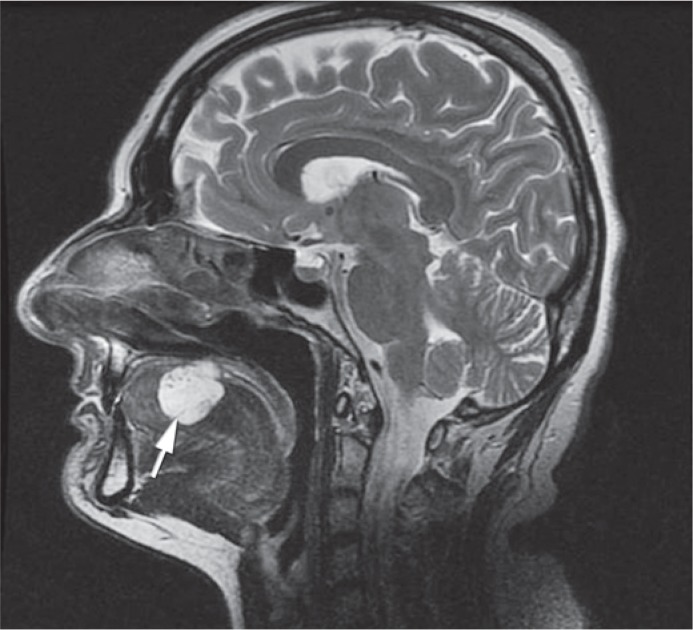
T2 weighted MR image in sagital plane before treatment shows 35 × 25 mm big lesion (white arrow) identified as submucosal cavernous haemagioma of the tongue. The lesion is expansive and does not involve the radix of the tongue.

**FIGURE 2.**
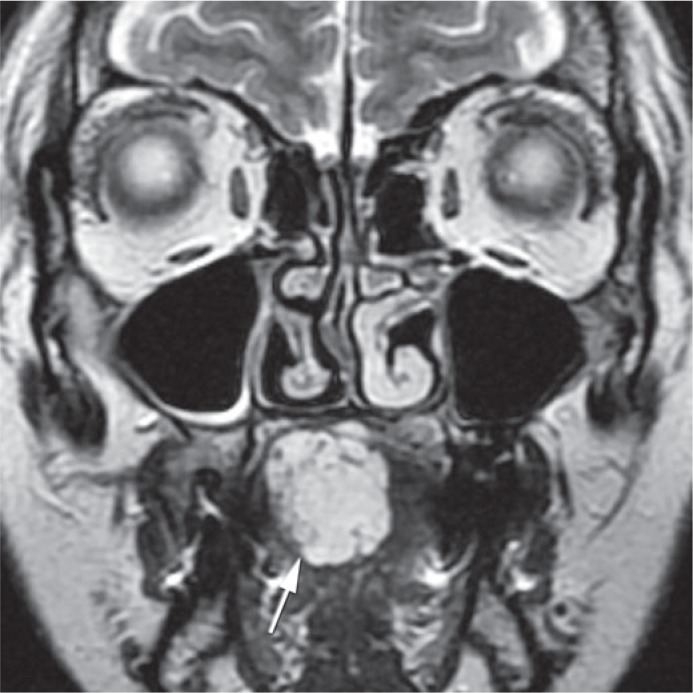
T2 weighted MR image of the same lesion (white arrow) in coronal plane before treatment shows that the majority of lesion is located on the left side of the tongue.

**FIGURE 3.**
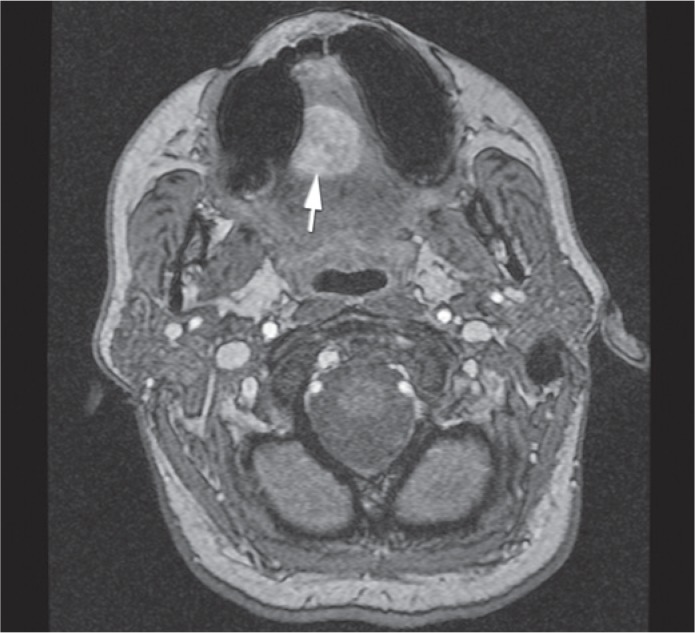
Axial plane of the lesion (white arrow) shown with MR angiography with time resolved imaging of contrast kinetics (TRICKS) before treatment.

Contrast enhanced MRI with time resolved imaging of contrast kinetics sequence (TRICKS) was performed, so that the best method of treatment could be selected (Figure 1, Figure 2 and Figure 3). The maxillofacial surgeon planed a surgery, so we were asked to perform a preoperative intra-arterial embolization. Because of the superficial localization of the lesion, we chose a direct puncture as the most appropriate approach.

The procedure was performed under general anaesthesia. This provided us the complete analgesia and prevention of tongue movement during the puncture and the injection of the ethanol and the contrast media.

**FIGURE 4.**
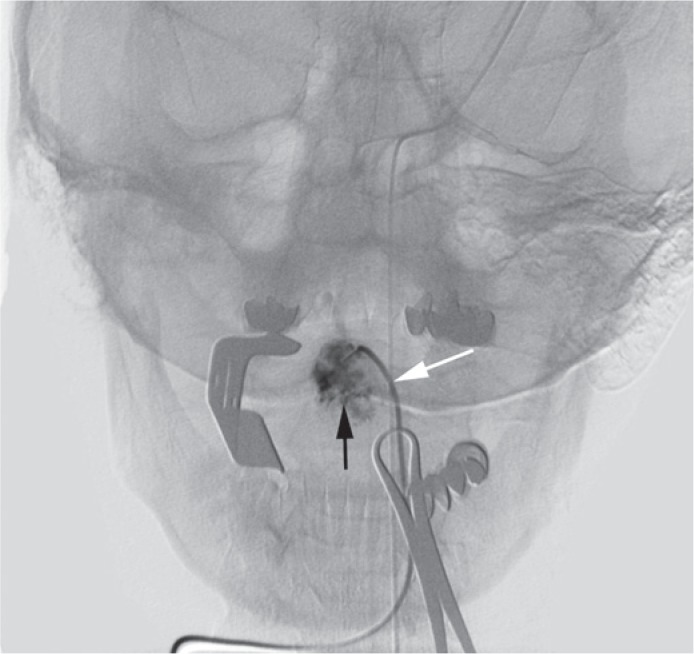
Digital, nonsubtracted, image of tongue parenchimography during sclerotization with opened mouth with distractor and cannula (white arrow) placed in haemangioma (black arrow).

**FIGURE 5.**
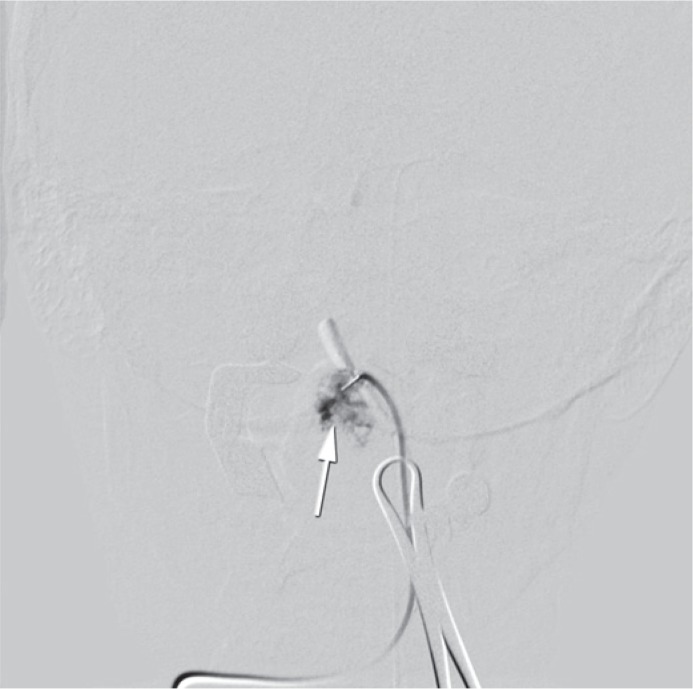
Digital subtractoinal image during the procedure enabled us a more controlled injection of ethanol and gave us a possibility to avoid the reflux to the venous side. The haemangioma of tongue is indicated with the white arrow.

**FIGURE 6.**
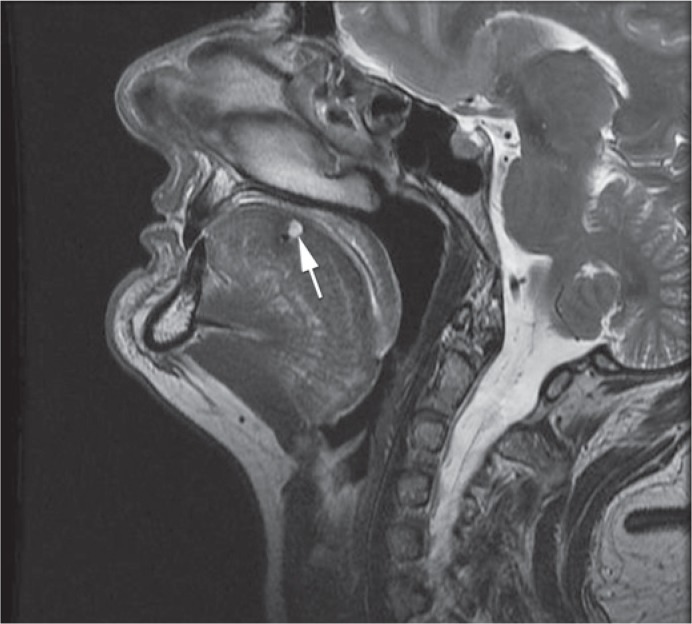
Control postcontrast sagital T2 weighted image of tongue, six months after the procedure, showing a small remnant of the cavernous haemangioma (white arrow).

We performed parenchimography of the lesion using several projections to reach an optimal view of the lesion and to evaluate the potential extravasation of contrast from haemangioma (Figure 4, Figure 5). This enabled us a more controlled injection of ethanol and gave us a possibility to avoid the reflux to the venous side. For the puncture we used 10 to 12 G needle (Terumo, Tokyo, Japan). After the puncture the proper position of the needle was verified with a continuous reflux of blood. Then the cannula was flushed with a 5% glucose solution. Under fluoroscopy control 96% alcohol diluted with Lipiodol (ratio 1:5) was injected. The cannula was removed and next puncture was performed. All together we performed six punctures and injected approximately 6 ml of ethanol. There were no technical complications during the procedure (Figure 3). No major bleeding was observed after the procedure. After the procedure the patient received analgetics and antioedematous therapy. After the sclerotization the planed surgery was abandoned. Control MRI examinations 6 and 12 months after the procedure showed only a small remnant of haemangioma and no signs of a larger relapse (Figure 6).

## Discussion

Most haemangiomas can be treated conservatively with an expectant strategy.^1^ However there are occasions when haemangiomas need more active intervention with sclerotization or preoperative embolization followed by surgery.^8^ The correct diagnosis have to be made and the decision to intervene has to be balanced against non-intervention and spontaneous involution. The outcome has to be considered and treatment with sclerosants should be limited to haemangiomas where the site is not of a major aesthetic concern. Surgical debulking has to be considered in the same context with regard to the long term effects. Surgery involving extensive scarring should be avoided if the alternative is awaiting natural resolution and lesser scarring.

Usually treatment of the tongue vascular abnormalities is performed by the intra-arterial catheterisation and embolization with particles or n-buthyl cianoacrilat (NBCA). Such a treatment has good results, but it is not suitable for all types of malformations.^9^ Intra-arterial endovascular embolization therapy is limited by the number of arteries, technical problems related to the difficult vascular anatomy or by the occurrence of vasospasm, which can lead to the failure of the procedure.

Direct puncture and the application of ethanol were initially described as an alternative method in cases where conventional intra-arterial catheter embolization was technically not possible.^9^,^10^ In such cases the direct injection of the sclerosing agent results in a higher level of devascularization compared to the intra-arterial embolization. The relative simplicity and good results have led to a wider implementation and application of the method. Absolute ethanol has been accepted as a new sclerosing agent associated with a substantial reduction of lesion recurrence, which induces denaturation of tissue protein, precipitating protoplasm and subsequent permanent obliteration of the vessel lumen.^11^

Sclerotization must be carried out slowly so that the amount of ethanol entering the haemangioma vascular bed can be maximized. The movement of the sclerosing agent to avoid the passing to the venous side must be constantly monitored.^7^,^12^,^13^

With our intervention we allowed a patient, which was intended to undergo a mutilant surgery with the resection of the tongue, a year and a half of normal life until now.

Direct puncture of the cavernous haemangioma and sclerotherapy with alcohol proved to be a safe and effective method to achieve devascularization of the lesion and postpone or even avoid surgical resection.

## Figures and Tables

**TABLE 1.** Classification of vascular abnormalities according to Mulliken and Glowacki

**Vascular abnormalities**	
Haemangiomas (tumour): Usually not present at birth Rapidly increase in size Involute F:M = 3:1 60% Head and Neck Most common tumour of infancy “strawberry naevus”	Vascular Malformations: Present at birth (may not be clinically apparent) Grow in proportion to body size Can degenerate Can hypertrophy (AVM)

**TABLE 2.** International Society for the Study of Vascular Anomalies Classification System

**Vascular (or vasoproliferative) Neoplasms**	**Vascular Malformations**
Infantile haemangioma	Slow-flow vascular malformations Capillary malformation Venous malformation Lymphatic malformation
Congenital haemangiomas RICH NICH	
Kaposiform haemangioendothelioma and tufted angiomas (with or without Kasabach–Merritt syndrome)	Fast-flow vascular malformations Arterial malformation Arteriovenous fistula Arteriovenous malformation
Spindle cell haemangioendothelioma	
Epithelioid haemangioendotheliomas	
Other rare haemangioendotheliomas (i.e., composite, retiform, and others)	Combined vascular malformations (various combination of the above)
Angiosarcoma	
Dermatologic acquired vascular tumors (i.e., pyogenic granuloma)	

RICH = rapidly involuting congenital haemangioma; NICH = noninvoluting congenital haemangioma
